# A retrospective review of CT pulmonary angiogram confirmed pulmonary emboli in COVID-19 patients admitted to Groote Schuur Hospital, Cape Town

**DOI:** 10.4102/sajr.v26i1.2280

**Published:** 2022-02-28

**Authors:** Petri Ahlers, Mariam Q. Said-Hartley

**Affiliations:** 1 Department of Radiology, Faculty of Health Sciences, University of Cape Town, Cape Town, South Africa

**Keywords:** COVID-19, pulmonary emboli, computed tomography pulmonary angiogram (CTPA), South Africa, anticoagulation, pregnant, TB, HIV

## Abstract

**Background:**

A high incidence of thromboembolic phenomena has been widely reported in patients with coronavirus disease 2019 (COVID-19) pneumonia. There is, however, a paucity of data detailing the incidence and characteristics of pulmonary emboli (PE) in COVID-19 patients in the South African setting.

**Objectives:**

To describe the incidence and characteristics of PE confirmed by CT pulmonary angiogram (CTPA) in patients with COVID-19 pneumonia admitted to a tertiary hospital in the Western Cape, South Africa.

**Methods:**

This was a retrospective-, descriptive study of all adult patients with COVID-19 pneumonia confirmed by polymerase chain reaction (PCR) undergoing CTPA for suspected PE while admitted to Groote Schuur Hospital. The study period was from 01 April 2020 to 30 September 2020.

**Results:**

The study cohort consisted of 116 patients, 59% being female, of whom 29% were pregnant or in the postpartum period. The median age for both genders combined was 49.5 years. The overall incidence of PE was 19%, with 20% in our subset of pregnant and postpartum patients. The majority (64%) of PE’s were reported as being segmental in anatomical location.

**Conclusion:**

The noteworthy cohort included patients with pulmonary tuberculosis (PTB), HIV as well as pregnant and postpartum patients. The overall incidence of PE was 19% with no significant differences in demographics, comorbidities or D-dimer levels between patients with or without PE. The importance of a high clinical index of suspicion together with the role of CTPA in diagnosing PE in hospitalised COVID-19 patients is emphasised.

## Introduction

The strong association between coronavirus disease 2019 (COVID-19) pneumonia and venous thromboembolism (VTE) has been well described.^1,2,3^ Venous thromboembolism and, specifically pulmonary emboli (PE), can have devastating outcomes if left untreated.^4^ A hypercoagulable state in patients with severe COVID-19 pneumonia increases the risk of thrombosis.^3^ Risk factors provoking VTE include bed-bound patients, vascular lines, advanced age, high body mass index (BMI) and underlying cardiovascular abnormalities.^4^

Evidence suggests that all hospitalised patients with COVID-19 pneumonia should receive prophylactic anticoagulation in the absence of contraindications.^1,2,3,5^ Yet, a surprisingly high incidence persists despite concurrent anticoagulation, also highlighting the need for different and more effective anticoagulation.^1,6,7,8^

Pulmonary embolism presents with non-specific signs and symptoms, making the diagnosis of PE clinically challenging.^4,9,10^ This is particularly true in those with hypoxic COVID-19 pneumonia, where there is considerable overlap between the systemic and respiratory symptoms associated with COVID-19 and those of PE.^11^ An elevated D-dimer level on hospital admission and sudden worsening in clinical condition in patients with confirmed COVID-19 pneumonia should raise the suspicion of PE and CT pulmonary angiogram (CTPA) should be considered.^3,5,11^ Imaging with CTPA remains the gold standard in the diagnosis of PE.^9,10^

This study aimed to describe the incidence and characteristics of CTPA confirmed PE in patients with COVID-19 pneumonia admitted to a tertiary hospital in the Western Cape, South Africa.

## Study design and methods

A retrospective-, descriptive single-centre study design was used. The study included all adult patients admitted to Groote Schuur Hospital (GSH) from 01 April 2020 to 30 September 2020 with confirmed COVID-19 pneumonia who underwent CTPA scans. This was defined by a positive reverse transcriptase polymerase chain reaction (RT-PCR) test on a nasopharyngeal swab for severe acute respiratory syndrome coronavirus 2 (SARS-CoV-2). The indication for CTPA in all patients was suspected PE.

Radiological images and reports on scans performed in the Division of Radiology, Groote Schuur Hospital are stored in the Picture Archiving and Communications System (PACS) database system. A detailed search using query builder (Philips, xiris 8.3.16) database search tool was used to access all CTPA reports/images on the PACS system for the study period. The search phrases ‘covid’, ‘SARS’, ‘corona’, ‘Cov 2’, ‘person under investigation (PUI)’ and ‘PCR’ were used to refine the search results.

The initial search results with these search phrases revealed 269 CTPA studies. The study investigators manually filtered all 269 CTPA studies, and those patients with a history of COVID-19 and confirmed positive RT-PCR SARS-CoV-2 results were included in the search criteria. All PUI patients with positive RT-PCR SARS-CoV-2 results were re-classified as COVID-19 and included in the study.

Of the 269 studies, a total of 116 CTPA studies satisfied the search criteria and were enrolled in the study. The included cases either had a confirmed RT-PCR SARS-CoV-2 result on the National Health Laboratory Service (NHLS) or a confirmed result documented on the CTPA clinical request form.

All CTPA studies were performed on a 160-slice Toshiba Aquillion PRIME (Tochigi, Japan) 160 multidetector computed tomography (MDCT) scanner. The scans were acquired with intravenous injection of 100 mL of contrast (Omnipaque 350), using a bolus tracking technique. The CTPA acquisition was initiated once the contrast bolus in the pulmonary trunk reached a Hounsfield unit (HU) of 180.

The CTPA images were transferred to the PACS database for viewing of both soft tissue and lung window reconstructions. All the CTPA studies were either reported by a Radiology consultant or reported by Radiology registrar, and the final report was approved by a Radiology consultant. The final CTPA report was used to confirm the presence or absence of a PE.

Pulmonary embolism was defined as a filling defect within the main pulmonary artery/arteries, and/or their lobar, segmental or subsegmental branches on the CTPA study.^9,10^ The anatomical location of the PE was documented. In the case of multiple PEs, the most proximal anatomical location was noted.

We further retrospectively collected data on patient demographics (age, gender), whether the patient was admitted to a general ward, high care or intensive care unit (ICU) and whether the patient had any recorded comorbidities on the clinical request form, such as diabetes, hypertension, cardiovascular disease, dyslipidaemia, HIV, malignancy, pulmonary tuberculosis, other respiratory diseases or a history of deep vein thrombosis (DVT).

Only D-dimer levels performed within three days of the CTPA study were included in the study. In a case where more than one D-dimer level was performed, the D-dimer result closest to the date of the CTPA study was recorded. In the absence of contraindications, all patients in the cohort received either prophylactic or therapeutic doses of enoxaparin according to our hospital protocol. As per hospital protocol at the time, therapeutic anticoagulation doses were reserved for patients who required high flow oxygen or mechanical ventilation, a high index of suspicion for PE or DVT, a rising D-dimer level on serial D-dimer measurements or a single D-dimer level > 1.5 times the upper limit of normal.^11^

### Statistical analysis

We recorded the incidence of CTPA confirmed PE in all patients with proven COVID-19 pneumonia with clinical suspicion for PE undergoing CTPA at tertiary hospital during the specified reporting period.

Furthermore, we investigated the association between demographic and clinical variables, and CTPA confirmed PE in patients with COVID-19 pneumonia. The appropriate non-parametric tests were conducted where the data violated assumptions of normality. Mann-Whitney U tests were used to compare age and D-dimer levels between COVID-19 patients with and without PE. Chi-square tests investigated the association between PE (positive or negative) and gender, anatomical location of PE, whether the patient was admitted to the ICU, high care or general ward, and whether the patient had comorbidities or not. Sub-analyses compared the female patients who were pregnant or within the postpartum period, versus those who were not pregnant.

Data was analysed using Statistical Package for the Social Sciences (SPSS) version 27, with the significance level set at *p* = 0.05.

### Ethical considerations

Ethical approval to conduct the study was obtained from the Faculty of Health Sciences Human Research Ethics Committee of the University of Cape Town (HREC reference number: 109/2021) and from Groote Schuur Hospital. All patient data were anonymised.

## Results

Of the study cohort of 116 patients, 69 (59%) were female and of these, 18 (26%) were pregnant and two (3%) were in the postpartum period. The median age for both genders was 49.5 years. Nineteen percent (19%) of patients undergoing CTPA for suspected PE had radiological confirmation of PE, with the majority (64%) reported as segmental in anatomical location.

A large proportion (85%) of the patients in the study were admitted to general wards, with 13% admitted to a high care unit and only 2% patients in ICU. (Table 1)

**TABLE 1:** Pulmonary emboli characteristics.

Pulmonary emboli characteristics	*N*	%
PE on CTPA	22	19
**Anatomical location of PE**		
Main	5	22.7
Lobar	3	13.6
Segmental	14	63.6
**Admitted to hospital**		
General ward	99	85.3
High care	15	12.9
ICU	2	1.7

*N* = 116.

PE, pulmonary emboli; CTPA, Computer Tomography Pulmonary Angiogram; ICU, intensive care unit.

The majority (71%) of patients in the cohort had comorbidities (Table 2), with hypertension and diabetes being the most common. Seventy two percent (72%) of patients with confirmed PE had more than one or multiple comorbidities compared to 47% in the non-PE group.

**TABLE 2:** Comorbidities.

Comorbidities	*N*	PE (*n* = 22)	Non-PE (*n* = 94)	*p*
Comorbidities	82	18	64	0.311
Patients with more than one comorbidity	43	13	30	0.067
Diabetes	27	6	21	0.832
Hypertension	42	10	32	0.449
Cardiovascular	10	4	6	0.094
Dyslipidaemia	6	1	5	1.00
Previous PTB	5	1	4	1.00
Active PTB	6	2	4	0.319
Other respiratory diseases†	6	2	4	0.319
HIV	22	6	16	0.423
Malignancy	4	0	4	1.00
DVT	3	1	2	0.471

*N* = 116.

PBT, pulmonary tuberculosis; PE, pulmonary emboli; DVT, deep vein thrombosis; HIV, human immunodeficiency virus.

† , Other respiratory diseases include asthma, chronic obstructive pulmonary disease and sarcoidosis.

There was no significant difference in demographic or clinical variables, including D-dimer levels between patients with and without PE (Table 3). The incidence and characteristics of PE in the pregnant/postpartum and the non pregnant patients are presented in Table 4. The pregnant and postpartum group was significantly younger compared to the non-pregnant females, and had fewer comorbidities.

**TABLE 3:** Clinical characteristics of cohort.

Characteristics of cohort	PE (*n* = 22)					Non-PE (*n* = 94)					*p*
	Median	IQR	*n*	%	mg/mL	Median	IQR	*n*	%	mg/mL	
Age	53.5	34.5–61	-	-	-	49	35–59	-	-	-	0.585
Female	-	-	14	63.6		-	-	55	58.5	-	0.659
D-dimer level†	-	0.50–5.13	14	-	1.05	-	0.39–1.00	47	-	0.61	0.142
Days between RT-PCR SARS-CoV-2 test and CTPA‡	13	5–19	21	-	-	17	5–31	85	-	-	0.297

IQR, interquartile range; PE, pulmonary emboli; RT-PCR, reverse transcriptase polymerase chain reaction; SARS-CoV-2, severe acute respiratory syndrome coronavirus 2; CTPA, computed tomography pulmonary angiogram.

† , D-dimer levels within 3 days of CTPA study;

‡ , In 10 patients the date of the RT-PCR SARS-CoV-2 test was not indicated on the CTPA request form.

**TABLE 4:** Pulmonary emboli characteristics between pregnant, postpartum and non pregnant patients.

Pulmonary emboli characteristics	Pregnant/postpartum (*n* = 20)				Non pregnant (*n* = 49)				*p*
	*n*	%	Median	IQR	*n*	%	Median	IQR	
PE on CTPA	4	20	-	-	10	20.4	-	-	0.969
**Anatomical location of PE**									0.161
Main	-	0	-	-	3	30	-	-	-
Lobar	1	25		-		0	-	-	-
Segmental	3	75	-	-	7	70	-	-	-
**Admitted to hospital**									0.126
General ward	20	100	-	-	40	81.6	-	-	-
High care	-	0		-	8	16.3	-	-	-
ICU	-	0	-	-	1	2	-	-	-
**Comorbidities**	10	50		-	40	72.5	-	-	0.008
Age	-	-	30	23.7–32.7	-	-	55	41–62	< 0.001

Note: Eighteen patients were pregnant, and 2 patients were within the immediate postpartum period.

PE, pulmonary emboli; CTPA, computed tomography pulmonary angiogram; ICU, intensive care unit; IQR, interquartile range.

## Discussion

The incidence of PE in hospitalised patients with COVID-19 pneumonia undergoing CTPA for clinical suspicion of PE at our institution was 19.0%. This is similar to the incidence of 23.9% reported in a meta-analysis by Kwee et al. (2021). Their pooled incidence included studies with similar study design, as well as similar indications for CTPA, and likewise only included studies with CTPA confirmed PE.^12^

The pooled incidence of those admitted to general wards was 23.9%, with a higher incidence (48.6%) of PE reported in patients admitted to ICU.^12^ Most of the patients in the studied cohort were admitted to general wards, with only 2.0% admitted to the ICU. The general assumption was that patients with severe COVID-19 pneumonia were admitted to either high care or ICU, leading us to postulate that the majority had non-severe COVID-19 pneumonia. Peripheral PE was the most frequent anatomical location reported in international studies.^12,13,14,15^ This study had similar findings, with segmental PE the most common anatomical location.

Interestingly, the mean age of the study participants was younger than that reported in similar study populations undergoing CTPA for suspected PE.^16,17^ We observed a female predominance, which further consisted of a small subset of young pregnant patients and patients within the postpartum period. This could potentially explain and partially account for the difference in age observed in this study compared to the other studies. Not surprisingly, the pregnant and postpartum group was significantly younger when compared to the non-pregnant females, and had less age-related comorbidities. Other study populations with similar study designs have not commented on pregnant or postpartum patients. It is unclear whether pregnant patients were excluded from these studies or whether there were no pregnant patients in their respective study populations.^12,17^

Pregnancy and the postpartum period in the general population are associated with an increased risk of VTE, especially in the immediate postpartum period.^18^ However, there is a paucity of published work on the incidence and characteristics of PE in pregnant patients with COVID-19 pneumonia. A literature review conducted by Czeresnia et al. reported no difference in the severity of COVID-19 infection or symptomatology in pregnant patients compared to the non-pregnant population.^19^ It is unclear whether, given the hypercoagulable state of pregnancy, COVID-19 pneumonia or interplay between these two entities could have contributed to PE in this subgroup of patients. Larger studies including post-mortem studies would need to be conducted to comment on the true underlying pathophysiology. The incidence of PE in the subset of pregnant and postpartum patients in this study was 20%.

The mean D-dimer levels in the PE group were higher than in the non-PE group, but this was not statistically significant. No significant differences in demographic features or comorbidities between patients with and without PE were found.

There was a median 13-day interval between positive RT-PCR results for SARS-CoV-2 and the diagnosis of PE on CTPA. In a study by S. Meiler et al., the majority of PE’s were detected on days 11–20 after symptom onset.^16^ In addition, a similar study to assess PE in COVID-19 patients undergoing CTPA reported a median of 14 days duration of symptoms prior to CTPA.^17^ This highlights the proposal for potentially using a lower threshold for requesting CTPA during the 2nd- and 3rd week of COVID 19 infection. Unfortunately, we did not collect information on the delay between the onset of symptoms to the time of CTPA, and only reported on the number of days from the RT-PCR CoV-2 test to CTPA. This precluded a direct comparison to the above mentioned studies with the assumption that symptom onset most likely preceded the date of RT-PCR CoV-2 test.^16,17^

Identifying a time period when patients are most at risk of developing PE during COVID-19 infection could alert the clinician to have a lower threshold for requesting a CTPA study. This information would be invaluable, especially in view of the clinical challenges created by symptom overlap between PE and severe COVID-19 pneumonia.

### Study limitations

- Sample size was limited by the retrospective nature of the study.
- Caution should be taken regarding the true incidence of PE in hospitalised COVID-19 patients, as only patients who underwent CTPA for suspicion of PE were included. The incidence reported does not reflect the true incidence in all hospitalised COVID-19 patients.
- All patients included in the study received at least prophylactic anticoagulation, as per our institution guidelines at the time. However, no distinction was made between prophylactic or therapeutic doses, nor were exact doses included in the analysis.
- A small subset of pregnant and postpartum patients was included in our study. Larger studies are required to comment on the true incidence and characteristics of PE in COVID-19 pregnant and postpartum patients.
- We only included D-dimer levels performed within three days of the CTPA which resulted in a smaller sample size compared to our overall cohort. This was especially true for the non-PE group.
- We further acknowledge that D-dimer cut off levels used in the non-pregnant population are not reliable in pregnancy.^18^

## Conclusion

According to our knowledge, this study is the first to describe the incidence and characteristics of PE confirmed on CTPA in hospitalised COVID-19 patients in South Africa. The incidence of PE in patients with COVID-19 pneumonia undergoing CTPA for suspected PE at our institution was 19% with the majority of PE’s reported as segmental in anatomical location. The study cohort was unique as it included patients with pulmonary tuberculosis (PTB), HIV, as well as a subset of pregnant and postpartum patients.

We report no significant differences in demographic features, comorbidities or D-dimer levels between patients with and without PE. Our observations highlight the importance of CTPA, together with a high clinical index of suspicion, in diagnosing PE in hospitalised COVID-19 patients at our institution.

Larger multi-centre studies exploring the characteristics of PE, together with laboratory markers and historical data to help identify a time interval during the course of COVID-19 infection, when patients are most at risk of developing PE, could help with clinical decision making and in refining local hospital protocols, especially in resource constrained settings.
